# The new administration in the USA: impact of policy changes in development assistance on low- and middle-income countries, with the example of Nepal

**DOI:** 10.1080/16549716.2025.2543103

**Published:** 2025-08-20

**Authors:** Deepak Paudel, Guenter Froeschl

**Affiliations:** a Nepal Public Health Association, Lalitpur, Nepal; b Center for International Health, LMU Munich, Munich, Germany; c Teaching and Training Unit, Institute of Infectious Diseases and Tropical Medicine at University Hospital, LMU Munich, Munich, Germany

**Keywords:** Development assistance in health, global health, Nepal, USAID, LMIC

## Abstract

Investing in health drives individual wellbeing, productivity, educational achievements, and economic growth. Collective global efforts have historically eradicated diseases like smallpox and are making progress against others, such as polio and malaria. In many low- and middle-income countries (LMICs), public health spending is insufficient, with per capita expenditure far below what is needed to deliver and sustain essential health services. High-income countries, such as the United States, are contributing to LMICs to bridge these gaps through bilateral and multilateral development assistance. The United States Government’s longstanding support in Nepal has helped to strengthen the health systems and resulted remarkable achievements. However, the new United States administration’s abrupt decisions to halt the development assistance and closure of most of the projects will significantly impact global health initiatives, jeopardize health gains, and pose a risk to global health security. This call-for-action urge stakeholders to evaluate the current and potential impact of these decisions and adopt a cohesive approach to maintain development cooperation. LMICs also must reassess their health investments and implement reforms to build resilient, self-sustaining health systems.

## Paper Context

**Main findings:** The decisions made by the new administration in the United States have posed substantial challenges to the health sectors of many low- and middle-income countries.

**Added knowledge:** Low- and middle-income countries must prioritize the sustainability and resilience of their health systems by allocating domestic revenue towards essential health services. Development assistance should be strategically leveraged to enhance health system infrastructure, capacity, and innovation.

**Global health impact for policy and action:** To safeguard and advance global health outcomes, there is an urgent need to establish a unified and sustainable framework for development assistance in health.

## Background

Investing in health not only enhances individual health and wellbeing but also drives productivity, educational accomplishments, and economic growth [1]. Health investment levels differ significantly between countries. For example, per capita health expenditure in the United States is $12,474, in Germany $6,191, and in Nepal $65 [2]. The COVID-19 pandemic highlighted shortcomings in the global agenda of strengthening health systems, enhancing surveillance mechanisms, and supporting health for everyone to achieve global health and prosperity. Historically, collective global efforts have eradicated smallpox, polio is at verge of eradication, malaria is being eliminated, and progress in combating diseases like Ebola is promising [3,4]. Providing quality healthcare for everyone around the globe is crucial for global peace, stability, safe migration, and prosperity [1,5].

## Importance of foreign aid in health

Foreign aid plays a critical role in supporting low- and middle-income countries (LMICs) which grapple with low per-capita income, limited public spending in health, and infrastructural gaps. Even modest aid flows can have substantial impacts, in settings where resources are scarce and needs are huge [6]. USAID-supported global health efforts have prevented over 91 million deaths—30 million among children – and driven a remarkable 32% decline in under-five mortality rates [7]. The abrupt dissolution of USAID, coupled with major aid cuts from other donors such as the UK’s 40% reduction of development assistance, threatens to create significant gaps in health financing. Despite notable contributions and accomplishments, the effectiveness and impact of aid remains uneven. Prioritizing sub-national, targeted, and result-focused investments is essential for maximizing effectiveness and ensuring equitable improvements [8].

## Spending in health and development assistance

In many LMICs, public health spending falls far short of meeting minimum health system needs. For instance, in 42 low-income countries, primarily in Africa and South Asia, public health spending per capita remains below $30 annually, far below the estimated $90 per capita needed to support essential workforce and commodities in these regions [9]. High-income countries often contribute to partially bridge these gaps through development assistance. The United States has been a significant contributor to health sector financing in numerous LMICs [10]. The assistance is mostly channeled through bilateral projects, government-to-government treasury funding, multi-country projects, and contributions to multilateral mechanisms like the Global Fund to Fight AIDS, Tuberculosis, and Malaria, and the Global Alliance for Vaccines and Immunizations. The United States also contributed through United Nations agencies such as the World Health Organization (WHO), United Nations Children’s Fund, United Nations Population Fund, United Nations Program on AIDS, and International Organization for Migration. These supports make a huge difference to many LMICs, but represent only a small fraction (<1%) of total United States government spending, and proportionally remains lower than the assistance from many other high-income countries against their gross national income [11–13].

In Nepal, the development assistance funding in the fiscal year 2023 by the United States government was $146 million, of which nearly $79 million was invested in the health sector [14]. These investments are part of global health diplomacy, and they help to keep the United States as a strategic development partner in Nepal. For over 70 years, the United States government’s generous support has significantly contributed to Nepal’s development in health, education, and infrastructure development. From laying the foundation of Nepal’s first university, Tribhuvan University, to establishing effective family planning programs, to eliminating malaria in the southern plains, the United States has played a crucial role [15]. The assistance has also been instrumental in introducing innovation, modernizing the government’s accounting system, and pioneering health sector reforms. The United States has used health diplomacy as a tool to support its wider foreign policy, helping build international partnerships, foster goodwill, and expand its global influence [16]. The decision on the cut of the development assistance could not only undermine the technical achievements, but also diminish the United States’ strategic position and influence in the landscape of partners of Nepal [17].

The policy changes of the new United States administration, which took place on 20 January 2025, include withdrawing the United States from the WHO [18], and initially issuing a stop-work order and eventually dissolution of the United States Agency for International Development (USAID). Similarly massive funding cuts of the United States President’s Emergency Plan for AIDS Relief (PEPFAR) [19], and the United States Centers for Disease Control and Prevention (CDC) will result in significant set-backs to public health programs globally [20]. These actions have impacted millions of individuals with health needs, thousands of health professionals, and hundreds of international and local organizations and their employees globally, and particularly in LMICs; and the threat is likely to reverberate also back to the interests of the American citizens [21].

## Development assistance framework for Nepal’s health sector

USAID was one of the oldest, most trusted, and largest donors to the health sector in Nepal. USAID’s support in Nepal was focused on health system strengthening, basic nutrition programs, infectious disease control, delivery of essential health services, equitable access to adolescent and reproductive health, combating HIV/AIDS, strengthening supply chain management systems, improving hygiene and sanitation facilities at schools and health facilities, supporting rehabilitation for the disabled, strengthening of health system monitoring, and execution of standardized population and health facility surveys [22]. In addition to health-focused interventions, USAID also supported programs such as improving air quality in the capital city Kathmandu, improving enrollment and early-grade reading for children, supporting localization of development initiatives, enhancing public financial management systems, and improving agriculture and biodiversity practices, all of which also brings about indirect health benefits. The assistance was governed by and aligned with the United States government’s Country Development and Cooperation Strategy, the Government of Nepal’s International Development Cooperation Policy 2019 [23], international agreements as set forth in the Paris Declaration on Aid Effectiveness in 2005 [24], the Accra Agenda for Action in 2008, the Busan Partnership for Effective Development Cooperation in 2011, as well as the Statement of Intent to Guide the Partnership for Health Sector Development in Nepal, the International Health Partnership for Universal Health Coverage 2030 [25], and Nepal’s National Health Sector Strategic Plan 2023–2030 [26]. These intertwined instruments showcase how the assistance was accountably governed, offering a balance of the priorities and interests of the governments both of the United States and Nepal.

Despite its economic levels and political unrest, impressive accomplishments in Nepal’s health sector over the last three decades (Table 1) were made possible through collaborative efforts between the government, development partners, non-governmental organizations, academia, and civil society [27]. These efforts were guided by a unified, country-led health strategy to which all stakeholders contributed, either through a pooled funding mechanism or harmonized projects under a single overarching strategy. However, the decision to abruptly close USAID projects will significantly impact the development landscape, including the public health programs funded by the government of Nepal and ultimately the access and quality of health services for the people of Nepal.

**Table 1.** Trajectory of key health indicators of Nepal, 1990–2020.

Year	1990	1995	2000	2005	2010	2015	2020
Life expectancy (yrs)	58	62	63	65	67	67	69
Total fertility rate	5.3	4.7	4	3.1	2.6	2.3	2.2
Under-five mortality rate	135.3	106.1	82.3	65.1	50.6	41.1	29.8
Maternal mortality ratio	850	539	504	380	349	252	174

Nepal’s health system remains highly vulnerable to shocks such as pandemics, natural disasters (e.g. earthquakes, landslides, and flooding), the (re)emergence of communicable diseases, and the increasing burden of non-communicable diseases, accidents and injuries, and mental health issues [28]. The COVID-19 pandemic and its aftermath threatened Nepal’s health gains by disrupting routine health service delivery, reducing the quality of care, and overstretching the health system with burden of communicable and non-communicable diseases [29,30]. The decision on foreign aid cuts is likely to severely affect resilience of Nepal’s health system and may risk losing some of the impressive gains Nepal has achieved over the last decades.

This decision will also have an immediate impact on the poorer stratum of the people of Nepal. Because of limited public spending on health, people in Nepal are currently paying 58% of the total health expenditure from their pockets. The decision will likely deprive the poorer stratum of people from accessing health services, potentially increasing their out-of-pocket expenses, and resulting catastrophic health expenditure [31]. As the United States government was generously funding and providing technical assistance to enhance quality care in both public and private sectors in Nepal, this decision may also disrupt such initiatives, leading to compromised and sub-optimal healthcare delivery as a result of limited supervision and quality monitoring mechanisms. As essential health services like immunization and nutrition are also facing challenges due to the reduction in funding, the government and development partners must urgently review and re-prioritize programs and their investments. As a result, the government has appealed to the United Nations and donors in the 78th World Health Assembly to continue the support for life-saving interventions and protecting health services for vulnerable populations [32].

While foreign aid is important and helpful for strengthening health systems, it can also create dependency and may divert country priorities. However, with thoughtful planning, funding cuts can be reframed as an opportunity for building a self-reliant health sector in many LMICs. It also demands enhancement of national capacity, health sector reforms, review of national priorities, and investments to mobilize domestic resources – ultimately paving the way toward resilient systems and improved health outcomes.

## Conclusion

Foreign aid is a crucial component of health sector financing and technical assistance in Nepal. The abrupt discontinuation of United States foreign aid poses a new threat globally by challenging the global health security measures. It also affects the lives of people and their access to quality health services. Countries should also utilize this moment as an opportunity to reform for a self-reliant and fit-for-purpose health system by allocating domestic revenue towards essential health services and using development assistance strategically to enhance infrastructure and innovation. We urge all stakeholders to proactively analyze the potential impact of the decisions by the United States administration in the immediate and long term, and to consider a more cohesive approach to prepare and enhance sustainable mechanisms of development cooperation.

## Data Availability

Not applicable, as this is a ‘Current Debate’ paper without any specific data other than those cited in the article.
